# Idiopathic Macular Hole: Algorithm for Nonsurgical Closure Based on Literature Review

**DOI:** 10.18502/jovr.v18i4.14555

**Published:** 2023-11-30

**Authors:** Ahmad M Mansour, Maurizio Parodi, Sami H Uwaydat, Suzanne Charbaji, Javier Ascaso, Hana A Mansour, Koushik Tripathy, Antônio Marcelo Barbante Casella

**Affiliations:** ^1^Department of Ophthalmology, American University of Beirut, Beirut, Lebanon; ^2^Department of Ophthalmology, University Vita-Salute Milan, Milan, Italy; ^3^Jones Eye Institute, University of Arkansas Medical School, Little Rock, Arkansas, USA; ^4^Charbaji Consultants, Beirut, Lebanon; ^5^Department of Ophthalmology, Hospital Clínico Universitario Lozano Blesa, Zaragoza, Spain; ^6^ASG Eye Hospital, Kolkata, West Bengal, India; ^7^Department of Ophthalmology, Estadual de Londrina, UEL, Londrina, PR, Brasil

**Keywords:** Cystoid Macular Edema, Epiretinal Membrane, Macular Hole, Posterior Vitreous Detachment, Vitreomacular Traction

## Abstract

Our purpose is to review the closure time and optical coherence tomography (OCT) biomarkers that result in the non-surgical repair of idiopathic full-thickness macular holes (IFTMH). Our methodology consisted of a comprehensive literature review of the nonsurgical resolution of IFTMH followed by the calculation of the estimated closure time using the structural equation model. Forty-nine studies were found eligible yielding 181 eyes with IFTMH: 81.1% being small holes ( < 250 µm) with a median diameter of 166 µm. Final vision (mean 20/41) was related to initial vision (mean 20/65) and mean age (67 years). The hole diameter was correlated with initial vision and closure time (mean 3.9 months). Closure time was related to hole diameter and initial vision in the following algorithm: Closure time (month)= - 0.057 + 0.008 diameter (µm) + 0.021 age (year) + 2.153 initial vision (logMAR). Biomarkers by OCT for self-closure included in decreasing frequency: pointed edge, de-turgescence of cystic macular edema (CME) with reversal of bascule bridge, and vitreomacular traction (VMT) release. The crucial function of Muller cell bridging in sealing the hole attests to its exceptional capacity for regeneration. After the hole has begun to close; however in less than 5%, a delayed restoration of the ellipsoid layer or a persistent outer foveal defect may prevent visual recovery and reopening of the hole is possible. In conclusion, eyes with small-size IFTMH and good baseline vision can have the additional option of close OCT monitoring for biomarkers of self-sealing biomarkers. When rehabilitative activity seems to be lacking, surgery is therefore mandatory.

##  INTRODUCTION

The concept of spontaneous remission of disease is widely known in both medicine and surgery, particularly in the field of oncology. There have been reports on both spontaneous rhegmatogenous retinal detachment resolution and spontaneous epiretinal membrane separation.^[1,2]^ It is well established that symptomatic idiopathic full-thickness macular hole (IFTMH) is treated surgically.^[3]^ Poorer anatomic and visual outcomes in eyes having vitrectomy are related to the longer duration of IFTMH symptoms.^[4]^ Hence, the time from patient presentation to surgery needs to be brought to a minimum.^[4]^ There remains a small subset of IFTMH that closes without surgery. In this article, we examine the most recent research to evaluate the OCT hallmarks that predict self-sealing of IFTMH and discuss how to advise clinicians to prevent delays in surgical intervention while still providing a threshold for self-restoration, particularly in specific groups of IFTMH.

##  METHODS

The peer-reviewed ophthalmic literature was searched in Google Scholar, PubMed, Embase, and Web of Science Core Collection databases and was last updated on July 17, 2023 by one of us (HAM). The search strategy used the terms “macular hole” and “spontaneous closure” with languages restricted to English, Spanish, French, German, and Italian. Chinese-language articles were also added based on Chinese reviews. Studies were included if they reported 1 or more cases of nonsurgical closure of IFTMH with a detailed description of age, gender, visual acuity on presentation and follow-up, as well as hole size. One of us (AMM) read all the original articles, assessed their eligibility, and extracted the data.

IFTMH size was considered the smallest inner diameter mentioned by the authors. IFTMH was classified according to the International Vitreomacular Traction Study Group as follows: small < 250 μm; medium 250–400 μm; and large > 400 μm. Inclusion criteria were IFTMH confirmed by spectral domain SD-OCT with periodic OCT exams until hole closure. Eyes that received topical therapy to help with hole closure were also included. Exclusion criteria: 1- Prior vitreoretinal surgery (including delayed spontaneous closure of FTMH following vitrectomy); 2- lamellar macular hole; 3-foveoschisis and retinoschisis; 4-macular degeneration; 5-sun gaze maculopathy, laser pointer maculopathy, drug-induced maculopathy, and macular telangiectasia; 6-chorioretinitis, ocular trauma, retinal detachment, high myopia, retinal vein occlusion, and retinitis pigmentosa; 7-pediatric age. One of us (AMM) measured the hole size based on horizontal OCT scans when information about the size was missing.

### Statistical Analysis 

We used SPSS 26 (SPSS, Inc., Chicago, IL) to perform statistical analyses. Snellen visual acuity was converted into logMAR. Variables were described using the mean and Standard Deviation (SD). Significant variables of the ANOVA test and Chi-square test were entered into multivariable logistic regression models. To come up with a formula that relates the various variables, one of us (SC) used the structural equation model with estimated path coefficients. A *p*-value less than 0.05 was considered statistically significant.

**Figure 1:**
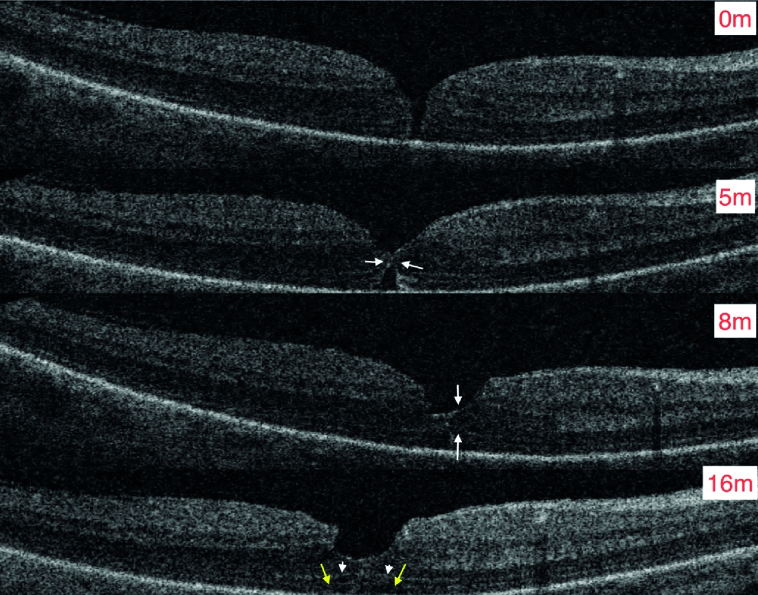
Healing by middle retina apposition (pointing edge) with middle retinal bridge thickening between month five (white arrows) and month eight (white arrow) with complete formation of ELM (arrowheads) and gradual maturation of EZ (yellow arrows) (month 16) (Courtesy of Alex Assi, MD). This is the right eye of a 48-year-old White man who presented with 20/40 best corrected visual acuity and after three years of follow-up visual acuity improved to 20/30.

**Figure 2:**
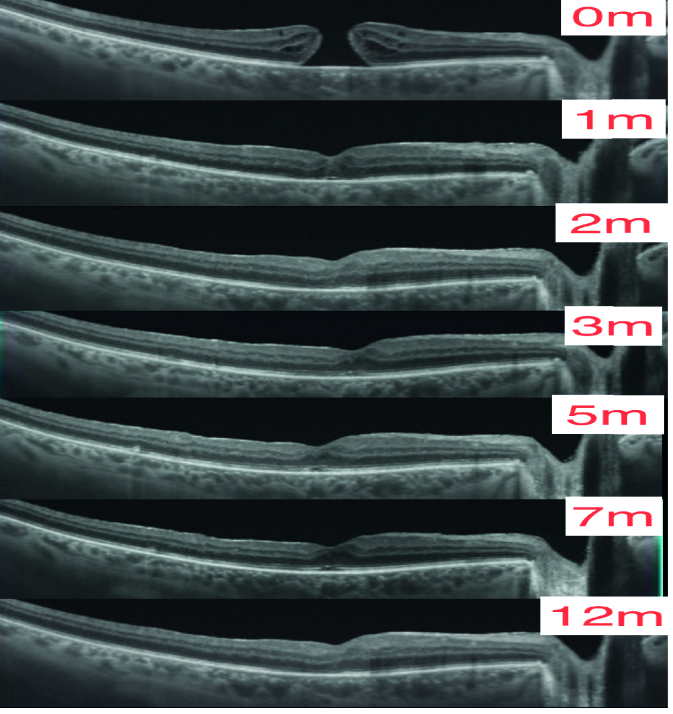
Healing by resolution of intraretinal cysts and reversal of bascule bridge leading to reapposition with no tissue loss (Courtesy of Luiz Lima, MD). This is the right eye of a 55-year-old Hispanic man followed over a period of one year. The hole closed one month after presentation. The ellipsoid zone was fully reconstituted one year after presentation.

**Figure 3:**
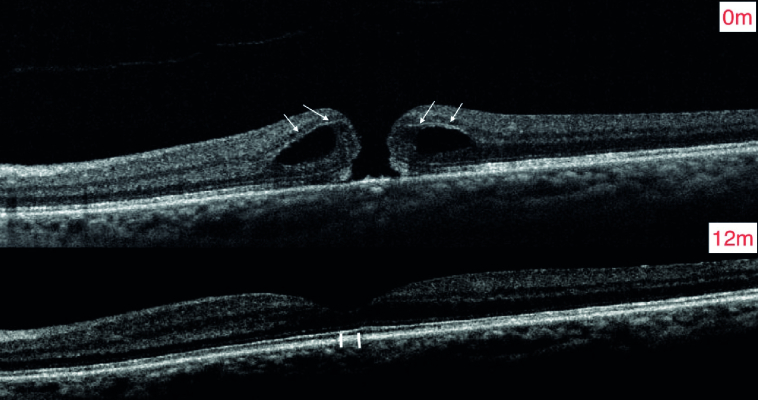
Reapproximation of the hole by hydration (giant cyst- arrows) decreasing the gap and allowing the hole to close and EZ reapproximation (arrowhead) (Courtesy of Raghav Ravani, MD). This 65-year-old Asian woman presented with visual loss of left eye to 20/60 (best corrected spectacle vision) and 196 µm macular hole 1.5 years after uneventful phacoemulsification with intraocular lens implant. She was placed on topical nonsteroidal anti-inflammatory and corticosteroid. There was loss due to follow-up during the COVID outbreak. One year later, visual acuity improved to 20/30 with a sealed macular hole.

**Table 1:** Reported rates by year of publication of nonsurgical closure of IFTMH confirmed by OCT.


orange**Author (year)**	orange**Country**	orange**Number of eyes**	orange**Prospective vs retrospective**	orange**Follow-up time (month)**	orange**Mean age**	orange**% closure**
Nawrocka ^[17]^ (2022)	Poland	750	Retrospective	5	67	23 (8.9%)
Ang ^[22]^ (2022)	Taiwan	124	Retrospective	19.5	58	3 (2.4%)
Dugel ^[18]^(2016)	USA	26	Prospective	24	NA	4 (15.4%)
Haller ^[19]^ (2015)	USA	47	Prospective	6	70.7	8 (17%)
Sugiyama ^[20]^ (2012)	Japan	142	Retrospective*	2	67.4	5 (2.7%)
Privat ^[21]^ (2007)	France	510	Retrospective*	2	70.7	14 (2.7%)
	
	
white<bcol>7</ecol>*awaiting surgery

**Table 2:** Clinical summary of nonsurgical closure of primary FTMH reported up to July 17, 2023 ${}^{\text{[17--56]}}$ .


Age, y (n = 181)	67.8 ± 7.4 (25-88; median 67)
Gender (n = 107)	M = 47 (43.9%)F = 60 (56.1%)
Hole Size, µm (n = 180)	185.6 ± 115.3 (40-698; median 166) Small ( < 250) = 146 (81.1%)Medium (250-400) = 27 (15.0%)Large ( > 400) = 7 (3.9%)
Initial BCVA, logMAR (n = 181)	0.515 ± 0.305 (range 0-2, median 0.54)
Final BCVA, logMAR (n = 181)	0.315 ± 0.277 (range 0-2, median 0.30)
Closure time, month (n = 180)	3.9 ± 8.1 (range 0.5-52, median 5)
Follow-up length, month (n = 181)	24.2 ± 33.0 (range 1-157, median 14)
	
	
white<bcol>2</ecol>BCVA, best corrected visual acuity; F, female; M, male; logMAR, logarithm minimum angle of resolution; Data reported as mean ± SD; The reference list was trimmed with bibliography including references not cited by Garg et al ^[24]^ and Liang et al ^[23]^.

**Table 3:** Estimated closure time calculated from a prediction equation based on initial vision and diameter: Closure Time (month) = - 0.057 + 0.008 Diameter (µm) + 0.021 Age (year) +2.153 Initial Vision (logMAR). This simulation tells us that a young person with a 100 µm diameter hole and initial vision of 20/25 can witness self-sealing of the hole within a month after observation while an elderly with the same hole parameter would need close to three months to heal.


orange**Age (year)**	orange**Diameter FTMH (µm)**	orange**Initial BCVA (logMAR)**	orange**Estimated closure time (month)**
20	100	0.1	1.3
20	200	0.1	2.2
20	200	0.4	2.9
20	250	0.7	3.9
20	250	1	4.5
90	100	0.1	2.8
90	200	0.1	3.6
90	200	0.4	4.3
90	250	0.7	5.3
90	250	1	6.0
	
	
white<bcol>4</ecol>*FTMH, full thickness macular hole; BCVA, best corrected visual acuity

**Table 4:** Cellular mechanisms of nonsurgical closure in decreasing order of frequency


	**Temporal sequence** ^[30,39,53]^
	A-Muller cells undergo de-differentiation
	B-These cells bring the hole's walls together
	C-Restoration of the external limiting membrane
	D- Thickening of the middle layer of the retina gradually into a more normal retina
	E-Reconstitution of the ellipsoid zone which is the last and slowest healing stage^[30,39,53]^
	**Biophysical Pathways**
	**1-Pointing edge configuration (tissue loss): **
	Thinning and centripetal elongation of the hole edge closes the gap (like a car antenna). This is the commonest pathway up to 90% of IFTMH^[41]^.
	**2-Dehydration theory with reversal of Drawbridge Maneuver (no tissue loss)**
	When the macula swells up, it has no place except to reach up the vitreous cavity with separation of the retina. Drying the macula will deterges the centrifugal forces and allows the macula to re-appose as there is no loss of tissue. Here there is a role for topical therapy CAI, NSAID, CST.^[33]^
	**3-VMT release**
	Release of traction allows the retina to settle initiating the re-apposition mechanisms.^[39,45,53]^
	**4-Swelling of edges (hydration theory) closes the gap (ET extraterrestrial finger) (tissue loss)**
	Spontaneous or induced swelling of the hole rim by coalescence of cystic cavities results in narrowing the gap with approximation of the boundaries and contact of the margins. Frank CME facilitates spontaneous closure by reapproximating the margins through physical expansion, hence the role of prostaglandin analogues.^[31]^
	
	
white<bcol>2</ecol>VMT, vitreomacular traction; CAI, carbonic anhydrase inhibitor; NSAID, nonsteroidal anti-inflammatory drug; CST, corticosteroid; CME, cystoid macular edema

### Demographics

IFTMH is reported to affect 0.02% of Americans and 0.16% of Chinese people, respectively.^[6,7]^ In Tasmania, Australia, Darian-Smith et al discovered an annual incidence of 4.1 per 100,000 people.^[8]^ The age- and gender-adjusted annual incidences of IFTMH were 7.9 and 7.4 per 100,000 inhabitants in Norway.^[9]^ The prevalence of bilateral FTMH was noted to be around 18%.^[10]^ In the US, females predominated between 1.66 and 2.6 times and by 2.2 times in Norway.^[7,9,11]^ (The Eye Disease Case-Control Study Group). The mean age (standard deviation) at presentation for IFTMH was 56.2 (9.2) in the US.^[7]^ In a previous literature review of 203 cases of IFTMH published in 2010, the mean age was 62.0 with 2.2 times female predilection.^[12]^

Most of the writing about macular holes focused on Caucasians and Asians. There are no pertinent reports on the black race. According to two US research and two UK studies, people of African descent tend to have bigger IFTMH sizes than people of Caucasian ancestry, which may be related in part to their delayed presentation, and longer wait times for surgery.^[13,14,15,16]^

### Incidence of Nonsurgical Closure

IFTMH closure rates were reported in various investigations to range from 2.4% to 17%.^[17,18,19,20,21,22,23,24]^ The cumulative closure rate for the prospective studies was 16% (12 of 73) [Table 1]. In a literature review of IFTMH,spontaneous closure rates werestratified by hole size: 22.2% for holes < 250 µm; 13.3% for 250–400 µm and 0% for > 400 µm.^[24]^

Spontaneous closure of bilateral IFTMH has been reported.^[25,26,27,28]^ Closure can be simultaneous or lagging by as much as two months. In bilateral FTMH, it is recommended to observe for two months before considering surgery if the fellow eye is sealed nonsurgically.

##  RESULTS

Forty-nine studies had complete data with a total of 181 eyes forming the basis of our analysis [Table 2].^[18][19][20][21][22][23][24][25,26,27,28][29][30][31][32][33][34][35][36][37][38][39][40][41][42][43][44][45][46][47][48][49][50][51][52][53][54][55][56]^ Mean vision improved significantly (*P* < 0.005) from 20/65 to 20/41 over a mean follow-up of two years. The median diameter of IFTMH was 166 with 81.1% being categorized as small holes. By multiple regression, the closure time correlated with initial vision (*P* = 0.014) and diameter (*P* = 0.015).

Final vision related to initial vision (*P* < 0.005) and age (*P* = 0.016). Hole diameter correlated with closure time (*P* = 0.013) and initial vision (*P* = 0.034). Visual gain related to shorter closure time (*P* = 0.039) and younger age (*P* = 0.029). Based on structural equation model, the prediction equation for closure time was the following:

Closure Time (month) = -0.057 + 0.008 Diameter (µm) + 0.021 Age (year) + 2.153 Initial Vision (logMAR).

##  DISCUSSION

Like the finding in juvenile FTMH,^[57]^ most of the crucial prognostic indicators for closure in our evaluation of adults with IFTMH was of small size. The majority of IFTMH that were sealed without vitrectomy were small macular holes. We proposed an algorithm to estimate the closure time based on hole diameter and baseline vision. This would guide clinicians in the clinical management of IFTMH. According to the formula, there is a delay of six days for self-sealing for every additional decade of life, for every EDTRS line loss (0.1 logMAR) and for every 25 µm increase in hole diameter. Besides, we advise close monitoring of OCT biomarkers for self-closure such as pointing edge morphology, resolution of CME or gradual relief of VMT. Patients with baseline good vision and small holes can be offered the choice of prompt intervention, surgically, or close monitoring if OCT findings offer the above favorable signs.

Muller cells are crucial in sealing the hole.^[30,39,53]^ Muller cells are dormant residential progenitor cells that have a great capacity for retinal neuron repair and functional plasticity. The initial step in achieving Müller glia dedifferentiation and proliferation is thought via activation of the Wnt signaling pathway. Thereafter, some Müller cells convert into neural progenitor cells replacing missing photoreceptors. The second cell involved in the repair of IFTMH is the retinal pigment epithelium (RPE). Retinal pigment epithelial cells contribute to spontaneous closure by histology by migrating onto the inner retina, proliferating, and dragging IFTMH margins centripetally.^[58]^ Twenty-two autopsy specimens with IFTMH were investigated by Guyer et al for their histopathologic characteristics; 16 (73%) had ERM, 15 (68%) had CME, and 3 (17%) were sealed by fibroglial and RPE hyperplasia.^[58]^

### Mechanisms of Hole Closure

Various mechanisms are detailed in Table 4, the commonest being the pointing edge. It remains that Müller cells are the key player in connecting the hole boundaries. Once contact occurs, the middle layer of the retina thickens and matures to form the external limiting membrane (ELM). The slowest phase of healing relies on reconstruction of the ellipsoid zone (EZ), and this is crucial for visual recovery.^[30,39,53]^

### Recurrence of IFTMH After Self-closure: A Sign of Delayed Healing 

Delayed healing forms a weak incomplete bridge of the hole susceptible to rupture from ocular rubbing or from centripetal ERM tractional forces or the new onset of VMT. The incidence is less than 5%.^[39,53]^ The eyes need to be constantly observed until the healing is fully mature. If the healing is slow or is halted, there is a considerable risk that the hole will reopen.

In conclusion, the decision to observe closely for spontaneous healing rather than immediate vitrectomy should be governed by the presence of a small diameter IFTMH, good initial BCVA, short duration of visual symptoms, minimal loss of tissue, and presence of 3 OCT biomarkers pointing edge configuration, gradual CME resolution following topical therapy and progressive release of VMT. A formula to prognosticate for spontaneous sealing is presented to guide the clinicians in their decision making.

##  Financial Support and Sponsorship

None

##  Conflict of Interest 

None.
